# Supplementary material for: Identification of Bulinus forskalii as a potential intermediate host of Schistosoma hæmatobium in Senegal
Source: PLoS Negl Trop Dis. 2023 May 9;17(5):e0010584. doi: 10.1371/journal.pntd.0010584 (PMC10198482; doi:10.1371/journal.pntd.0010584)
Supplement: S1 Table — (DOCX) [file pntd.0010584.s001.docx]

Supplementary data **S1 Table**

| Family | Species | Parts used | Storage method | Number of spectra in database (n=64) | Collection sites | Coordinates |
| --- | --- | --- | --- | --- | --- | --- |
| *Viviparidae* | *Bellamya unicolor* | Foot | Frozen | 2 | Richard-Toll, Senegal | 16˚27 N/15˚42 W |
| *Planorbidae* | *Biomphalaria pfeifferi* | // | Frozen | 6 | // | // |
|  |  |  | Ethanol | 16 | // | // |
| *Bulinidae* | *Bulinus forskalii* | // | Frozen | 4 | // | // |
|  |  |  | Ethanol | 2 | // | // |
|  | *Bulinus senegalensis* | // | Ethanol | 1 | Niakhar, Senegal | 14°30 N/16°30 W |
|  | *Bulinus truncatus* | // | Frozen | 3 | Richard-Toll, Senegal | 16˚27 N/15˚42 W |
|  |  |  | Ethanol | 24 | // | // |
|  | *Cleopatra bulimoides* | // | Frozen | 1 | // | // |
| *Lymnaeidae* | *Lymnaea natalensis* | // | Frozen | 3 | // | // |
| *Thiaridae* | *Melanoides tuberculata* | // | Frozen | 2 | // | // |

//: Same data
