# Supplementary material for: Identification of Bulinus forskalii as a potential intermediate host of Schistosoma hæmatobium in Senegal
Source: PLoS Negl Trop Dis. 2023 May 9;17(5):e0010584. doi: 10.1371/journal.pntd.0010584 (PMC10198482; doi:10.1371/journal.pntd.0010584)
Supplement: S2 Table — (DOCX) [file pntd.0010584.s002.docx]

Supplementary data **S2 Table**

|  | Samples names | Snail species ID by MS | Score Blind test with database | Result Shedding test | Result RT-PCR (Ct values) |
| --- | --- | --- | --- | --- | --- |
| 1 | KABfI1 | *Bulinus*_*forskalii*_Frozen_foot_senegal | 2.55 | (-) | (-) (n/a) |
| 2 | KABfI2 | *Bulinus*_*forskalii*_Frozen_foot_senegal | 2.69 | (-) | (-) (n/a) |
| 3 | KABfI3 | *Bulinus*_*forskalii*_Frozen_foot_senegal | 2.62 | (+) | (+) (34.13) |
| 4 | KABfI4 | *Bulinus*_*forskalii*_Frozen_foot_senegal | 2.37 | (+) | (+) (29.43) |
| 5 | KABfI5 | *Bulinus*_*forskalii*_Frozen_foot_senegal | 2.60 | (+) | (+) (29.23) |
| 6 | KABfNI1 | *Bulinus*_*forskalii*_Frozen_foot_senegal | 1.89 | (-) | (-) (n/a) |
| 7 | KABfNI2 | *Bulinus*_*forskalii*_Frozen_foot_senegal | 2.65 | (+) | (+) (34.86) |
| 8 | KABfNI3 | *Bulinus*_*forskalii*_Frozen_foot_senegal | 2.12 | (-) | (-) (n/a) |
| 9 | KABfNI5 | *Bulinus*_*forskalii*_Frozen_foot_senegal | 1.89 | (-) | (+) (28.57) |
| 10 | KABfNI7 | *Bulinus*_*forskalii*_Frozen_foot_senegal | 2.62 | (-) | (-) (n/a) |
| 11 | S20BfI6 | *Bulinus*_*forskalii*_Frozen_foot_senegal | 2.57 | (-) | (+) (33.05) |
| 12 | S20BfI7 | *Bulinus*_*forskalii*_Frozen_foot_senegal | 2.55 | (+) | (+) (23.01) |
| 13 | S20BfI8 | *Bulinus*_*forskalii*_Frozen_foot_senegal | 2.13 | (-) | (-) (38.30) |
| 14 | S20BfI9 | *Bulinus*_*forskalii*_Frozen_foot_senegal | 2.56 | (+) | (+) (31.17) |
| 15 | S20BfI10 | *Bulinus*_*forskalii*_Frozen_foot_senegal | 2.63 | (+) | (+) (25.00) |
| 16 | KSD2BfI1 | *Bulinus*_*forskalii*_Frozen_foot_senegal | 2.29 | (+) | (+) (19.39) |
| 17 | KSD2BfI2 | *Bulinus*_*forskalii*_Frozen_foot_senegal | 2.30 | (-) | (-) (n/a) |
| 18 | KSD2BfI4 | *Bulinus*_*forskalii*_Frozen_foot_senegal | 2.38 | (+) | (+) (25.41) |
| 19 | S20BfNI2 | *Bulinus*_*forskalii*_Frozen_foot_senegal | 2.36 | (-) | (-) (n/a) |
| 20 | S20BfNI6 | *Bulinus*_*forskalii*_Frozen_foot_senegal | 2.48 | (-) | (-) (37.50) |
| 21 | S20BfNI8 | *Bulinus*_*forskalii*_Frozen_foot_senegal | 2.43 | (-) | (-) (n/a) |
| 22 | S20BfNI9 | *Bulinus*_*forskalii*_Frozen_foot_senegal | 2.51 | (+) | (+) (34.37) |
| 23 | S20BfNI10 | *Bulinus*_*forskalii*_Frozen_foot_senegal | 2.26 | (-) | (-) (n/a) |
| 24 | MBOBfNI2 | *Bulinus*_*forskalii*_Frozen_foot_senegal | 2.32 | (-) | (-) (n/a) |
| 25 | MBOBfNI5 | *Bulinus*_*forskalii*_Frozen_foot_senegal | 2.35 | (+) | (+) (32.45) |
| 26 | KSD2BfNI1 | *Bulinus*_*forskalii*_Frozen_foot_senegal | 2.31 | (-) | (-) (n/a) |
| 27 | KSD2BfNI3 | *Bulinus*_*forskalii*_Frozen_foot_senegal | 2.18 | (-) | (-) (n/a) |
| 28 | KSD2BfNI4 | *Bulinus*_*forskalii*_Frozen_foot_senegal | 2.20 | (+) | (+) (28.84) |
| 29 | KSD2BfNI5 | *Bulinus*_*forskalii*_Frozen_foot_senegal | 2.28 | (-) | (-) (n/a) |
| 30 | KSD2BfNI7 | *Bulinus*_*forskalii*_Frozen_foot_senegal | 2.37 | (-) | (-) (n/a) |
| 31 | KSD2BfNI8 | *Bulinus*_*forskalii*_Frozen_foot_senegal | 2.27 | (-) | (-) (n/a) |
| 32 | KSD2BfNI10 | *Bulinus*_*forskalii*_Frozen_foot_senegal | 2.41 | (-) | (-) (n/a) |
| 33 | S9BfNI1 | *Bulinus*_*forskalii*_Frozen_foot_senegal | 2.31 | (-) | (+) (34.94) |
| 34 | SOBfNI1 | *Bulinus*_*forskalii*_Frozen_foot_senegal | 2.11 | (-) | (+) (33.88) |
| 35 | SOBfNI4 | *Bulinus*_*forskalii*_Frozen_foot_senegal | 1.87 | (-) | (-) (n/a) |
| 36 | SOBfNI5 | *Bulinus*_*forskalii*_Frozen_foot_senegal | 2.17 | (-) | (-) (n/a) |
| 37 | SOBfNI6 | *Bulinus*_*forskalii*_Frozen_foot_senegal | 1.82 | (-) | (-) (n/a) |
| 38 | SOBfNI7 | *Bulinus*_*forskalii*_Frozen_foot_senegal | 2.04 | (-) | (-) (n/a) |
| 39 | SOBfNI8 | *Bulinus*_*forskalii*_Frozen_foot_senegal | 2.10 | (-) | (-) (39.15) |
| 40 | MBOBfNI1 | *Bulinus*_*forskalii*_Frozen_foot_senegal | 2.22 | (-) | (-) (n/a) |
| 41 | MBOBfNI3 | *Bulinus*_*forskalii*_Frozen_foot_senegal | 2.28 | (-) | (-) (n/a) |
| 42 | MBOBfNI4 | *Bulinus*_*forskalii*_Frozen_foot_senegal | 2.18 | (+) | (+) (33.81) |
| 43 | MBOBfNI6 | *Bulinus*_*forskalii*_Frozen_foot_senegal | 2.34 | (-) | (-) (n/a) |
| 44 | MBOBfNI7 | *Bulinus*_*forskalii*_Frozen_foot_senegal | 2.41 | (-) | (-) (n/a) |
| 45 | SOBfNI2 | *Bulinus*_*forskalii*_Frozen_foot_senegal | 2.31 | (-) | (-) (n/a) |
| 46 | SOBfNI3 | *Bulinus*_*forskalii*_Frozen_foot_senegal | 2.30 | (-) | (-) (n/a) |
| 47 | SOBfNI9 | *Bulinus*_*forskalii*_Frozen_foot_senegal | 2.31 | (-) | (-) (n/a) |
| 48 | S5MDBfNI1 | *Bulinus*_*forskalii*_Frozen_foot_senegal | 2.10 | (-) | (-) (n/a) |
| 49 | S5MDBfNI2 | *Bulinus*_*forskalii*_Frozen_foot_senegal | 2.03 | (-) | (-) (n/a) |
| 50 | S17Bf17-1 | *Bulinus*_*forskalii*_Frozen_foot_senegal | 2.22 | (-) | (-) (n/a) |
| 51 | S17Bf17-2 | *Bulinus*_*forskalii*_Frozen_foot_senegal | 2.37 | (-) | (-) (n/a) |
| 52 | S17Bf17-3 | *Bulinus*_*forskalii*_Frozen_foot_senegal | 2.39 | (-) | (-) (n/a) |
| 53 | S17Bf17-4 | *Bulinus*_*forskalii*_Frozen_foot_senegal | 2.38 | (-) | (-) (n/a) |
| 54 | S17Bf17-5 | *Bulinus*_*forskalii*_Frozen_foot_senegal | 2.34 | (-) | (-) (n/a) |
| 55 | S17Bf17-6 | *Bulinus*_*forskalii*_Frozen_foot_senegal | 2.38 | (-) | (-) (38.89) |

(-) : Cercarial shedding test or Real Time-PCR : (+), positive ; (-), negative.

(n/a) : not available, because negative Real Time-PCR test with no Ct value
