# Supplementary figures and images for: Identification of Bulinus forskalii as a potential intermediate host of Schistosoma hæmatobium in Senegal
Source: PLoS Negl Trop Dis. 2023 May 9;17(5):e0010584. doi: 10.1371/journal.pntd.0010584 (PMC10198482; doi:10.1371/journal.pntd.0010584)

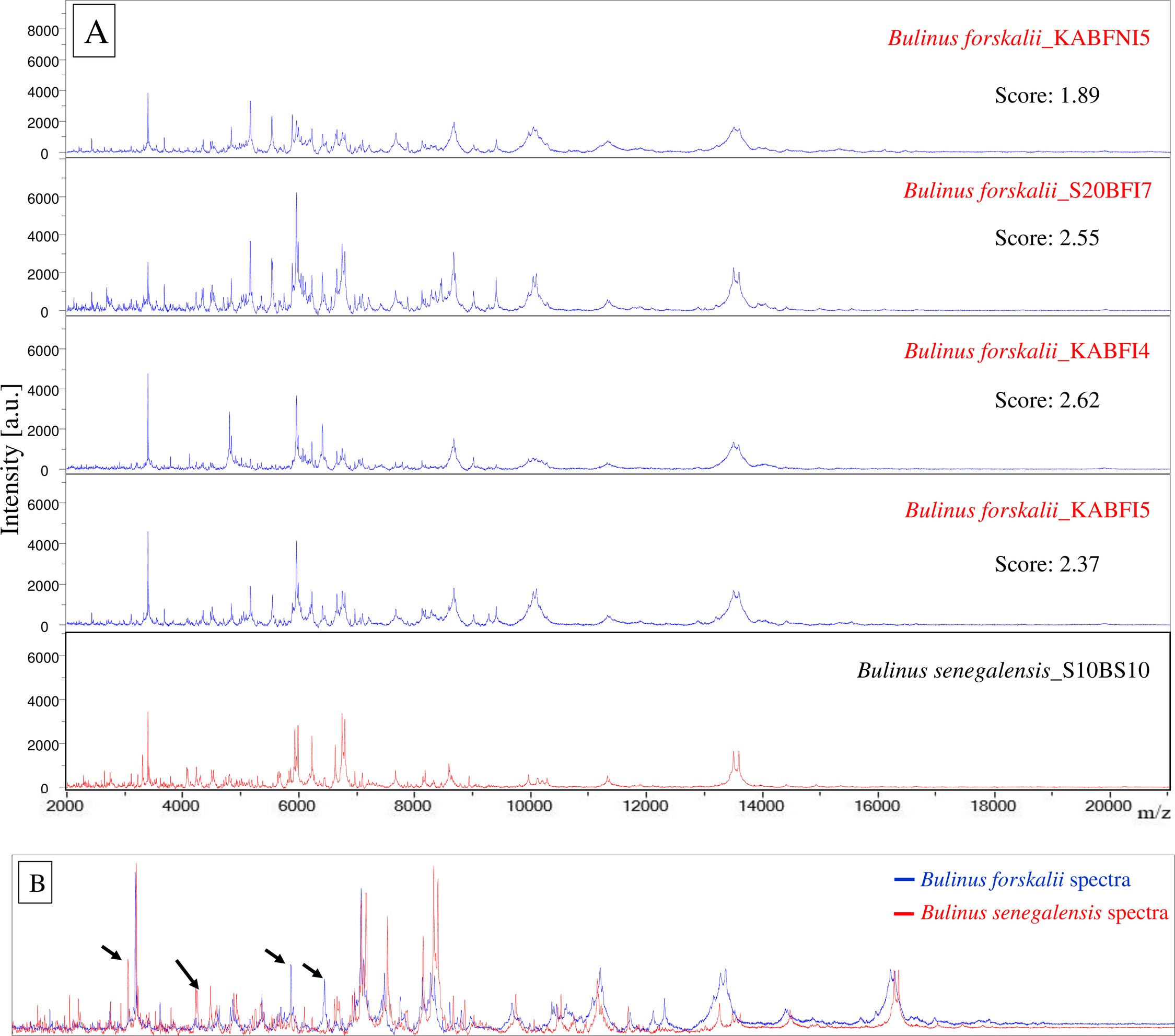

Supplement: S1 Fig — (A) Distinction of MALDI-TOF MS spectra of Bulinus forskalii (blue) and Bulinus senegalensis (red), illustrated by the spectral profiles of some specimens. a.u.: arbitrary units; m/z: mass/charge ratio. (B) Superimposed spectra of Bulinus forskalii (blue) and Bulinus senegalensis (red) with visually observed discriminating peaks indicated by black arrows. (TIF) [file pntd.0010584.s003.tif]
