# Supplementary material for: Identification of Bulinus forskalii as a potential intermediate host of Schistosoma hæmatobium in Senegal
Source: PLoS Negl Trop Dis. 2023 May 9;17(5):e0010584. doi: 10.1371/journal.pntd.0010584 (PMC10198482; doi:10.1371/journal.pntd.0010584)
Supplement: Attachment — Submitted filename: Responses to Reviewers 23.09.22.docx [file pntd.0010584.s004.docx]

Reviewer's Responses to Questions

<b>Key Review Criteria Required for Acceptance?</b></br></br>

As you describe the new analyses required for acceptance, please consider the following:</br></br>

<b>Methods</b></br></br>

-Are the objectives of the study clearly articulated with a clear testable hypothesis stated?</br>

-Is the study design appropriate to address the stated objectives?</br>

-Is the population clearly described and appropriate for the hypothesis being tested?</br>

-Is the sample size sufficient to ensure adequate power to address the hypothesis being tested?</br>

-Were correct statistical analysis used to support conclusions ?</br>

-Are there concerns about ethical or regulatory requirements being met?</br></br>

Reviewer #1: (No Response)

Reviewer #2: -Are the objectives of the study clearly articulated with a clear testable hypothesis stated?

The objectives and hypothesis have been implicitly stated. These could be explicitly stated in an additional paragraph in the introduction.

**Response 1** : We agree with reviewer’s comment and have added sentence in the introduction section of the manuscript – (Line 91)

-Is the study design appropriate to address the stated objectives?

Yes.

-Is the population clearly described and appropriate for the hypothesis being tested?

While the study focuses on *B. forskalii*, inclusion and/or discussion of other snail species found in the field sampling area would strengthen the manuscript.

**Response 2** : We agree with the reviewer's comment. Other species of snails were found in the sample area but data on them are already the subject of another paper in progress.

-Is the sample size sufficient to ensure adequate power to address the hypothesis being tested?

Yes, assuming the hypothesis is something to the effect of "*B. forskalii* snails that reside in the delta region of the Senegal River can carry *S. haematobium* infections". The current study conducted experiments on a smaller scale in terms of geographical location compared to other referenced studies of intermediate hosts of Schistosoma parasites.

-Were correct statistical analysis used to support conclusions?

No statistical analyses were described. Perhaps there could be a test to correlate cercarial shedding to Ct value or COX1 and ITS2 (yes/no) status.

**Response 3** : Thank you for your suggestion, the correlation between cercarial excretion and Ct value would be relevant but we did not count the number of cercariae excreted by each specimen.

-Are there concerns about ethical or regulatory requirements being met?

No, there are no ethical or regulatory concerns.

-Other comments

Consider adding information to the methods section to answer the following questions: How was the cercarial shedding test done? How was the DNA extracted? How was the RT-PCR conducted ? How was the PCR sequencing conducted?

**Response 4** : We agree with the reviewer's comment and have corrected them in the revised manuscript - (Line 120/125 ; Line 163/169 ; Line 170/178 and Line 183/205). A figure was also made to illustrate the emission test (fig 2.)

Reviewer #3: P.M. Gaye et al build on their previous work from 2021 where they use MALDI-TOF MS to identify host snails. Here they collect 55 Bu forskalli from the Senegal river delta and look for evidence of S.haemotobium infection. The methods section of the paper is not sufficient to describe their study, but can be easily improved upon.

**Response 5** : We agree with the reviewer's comment and have described the methods section of the manuscript in more detail.

1) Please describe in more detail the area of the delta where the *Bu forskalli* was collected; a map may be helpful here.

**Response 6** : We have corrected this in the manuscript – (Line 95/105) – (Fig 1)

2) Include in your methods how the snails were held (under what conditions) until the experiment to determine the presence of Sh or other parasites.

**Response 7** : Thanks for the remark, this part has been added in the manuscript– (Line 106/125)

3) Describe your shedding protocol. How were the cercariae induced from the *Bu forskalli*?

**Response 8** : Thanks for the remark, this part has been added in the manuscript – (Line 121/125) (fig 2.)

4) On line 75, the authors tell us they used a reference spectra database and provide the link. Please describe this database as it was not intuitive and this Reviewer could not find the relevant page for snail references.

**Response 9** : We agree with the reviewer's comment, we have added a description of the database in the revised manuscript along with a table - (Line 148/155).

We have also updated the link to access the database in the manuscript

--------------------

<b>Results</b></br></br>

-Does the analysis presented match the analysis plan?</br>

-Are the results clearly and completely presented?</br>

-Are the figures (Tables, Images) of sufficient quality for clarity?</br></br>

Reviewer #1: Improve figure 1 quality.

**Response 10:** Corrections have been made as recommended

Reviewer #2: -Does the analysis presented match the analysis plan?

Yes.

-Are the results clearly and completely presented?

As suggested below, more of the data could be made available, such as the results for all 55 *B*. *forskalii* specimens in Table 1.

**Response 11** : We agree to the reviewer’s comment, we added this information in Supplementary data S1 table**.**

-Are the figures (Tables, Images) of sufficient quality for clarity?

Yes. See below for suggestions.

Figure 2: If possible, consider showing (or explicitly labeling) the MALDI-TOF MS spectra of the reference sample for *B. forskalii* and, if available, those of other *Bulinus* members. Also consider showing the corresponding log score values for each spectra.

**Response 12 :** We agree with the reviewer's comment, the corrections were made as recommended. - (Fig 3)

Table 1: The text describes 55 samples, while this table shows 10. How were these chosen? Are these specimens that met the Ct cutoff of 35 for the RT-PCR? Consider clarifying this in the text and/or caption. These results also do not show completely uninfected specimens, that is, specimens with a negative shedding test, CT>35, and negative sequencing results for COX1 and ITS2. Perhaps there were some, but they are not shown in the table? Consider including the data for all tested specimens, if not in this table, as supplementary information.

**Response 13 :** We agree to the reviewer’s comment. However these 10 samples are the RT-PCR positive samples with a Ct less than 35. We have clarified this in the Materials and methods section ("Real-time PCR and PCR Sequencing") (Line 171/205)

All shedding test positive samples were also RT-PCR positive. Only 17 (55 samples) were RT-PCR positive with Ct less than 35. These 17 *S. haematobium* complex positive samples are of interest to us because the negative samples are not expected to give sequencing results (COX1 and ITS). Knowing that *S. haematobium* and S. bovis are part of the *haematobium* group.

-Other comments

On line 67, the manuscript states that "[the authors] collected (n=55) snails from the Senegal River Delta area, identified as *B. forskalii*". Were there other *Bulinus* snail species present but not considered for analysis? While perhaps outside the scope of the study, such information might help describe the contribution of B. forskalii relative to other *Bulinus* species as an intermediate host for *S. haematobium*.

**Response 14 :** We agree with the reviewer's comment but the other *Bulinu*s species collected will be the subject of another publication in which the aspect concerning the contribution of B. *forskalii* as an intermediate host of *S. haematobium* will be more detailed.

Reviewer #3: The authors claim in their results that the *Bu forskalli* were identified using MALDI-TOF MS, but also confirmed by COX-Folmer. Can they include that data in this manuscript?

**Resonse 15** : We thank you for your comment. This data has been included in the manuscript (line 128/155 and line 201/205).

--------------------

<b>Conclusions</b></br></br>

-Are the conclusions supported by the data presented?</br>

-Are the limitations of analysis clearly described?</br>

-Do the authors discuss how these data can be helpful to advance our understanding of the topic under study?</br>

-Is public health relevance addressed?</br></br>

Reviewer #1: (No Response)

Reviewer #2: -Are the conclusions supported by the data presented?

Yes.

-Are the limitations of analysis clearly described?

Yes.

-Do the authors discuss how these data can be helpful to advance our understanding of the topic under study?

There could be more discussion about how this new knowledge that B. forskalii can carry S. haematobium infection can help improve epidemiological studies, surveillance efforts, and contribute to controlling schistosomiasis. For example, in surveillance studies, have researchers previously dismissed the possibility that B. forskalii snails could carry S. haematobium, and thus would assess a region to be at low risk of S. haematobium infections if only B. forskalii were found? If so, the findings in this study would encourage researchers to re-evaluate the guidelines for this hypothetical assessment.

**Resonse 16** : We agree with the reviewer's comment, this point has been discussed a little in the discussion section of the manuscript

-Is public health relevance addressed?

Yes, but, as suggested above, it could be more thoroughly discussed.

**Resonse 17** : We agree with the reviewer's comment, we have added more response elements in the manuscript showing the relevance of public health as recommended.

-Other comments

On line 112, the text asserts "Our findings demonstrate that B. forskalii can be parasitised by S. hæmatobium complex fluke, in particular, S. hæmatobium s.s., S. bovis s.s., and hybrids between them." The results do not necessarily demonstrate that S. haematobium x S. bovis hybrid species were detected in these snails. As stated on line 124, "discrepant ITS and COX-based identification in a given snail specimen can be interpreted either as an infection with one hybrid parasite or a co-infection with two distinct parasite species." Consider slightly modifying the statement to the effect of "Our findings demonstrate that B. forskalii can be parasitised by S. hæmatobium complex fluke, in particular, S. hæmatobium s.s., S. bovis s.s., and possibly S. haematobium x S. bovis hybrids."

**Resonse 18** : We agree the reviewer's comment and added modifications in the discussion section of the manuscript – (Line 234-236)

Reviewer #3: The authors' conclusions are supported by the data presented.

--------------------

<b>Editorial and Data Presentation Modifications?</b></br><br/>

Use this section for editorial suggestions as well as relatively minor modifications of existing data that would enhance clarity. If the only modifications needed are minor and/or editorial, you may wish to recommend “Minor Revision” or “Accept”.

Reviewer #1: Introduction:

There is some data of B. forskalii and B. senegalensis distribution in Senegal? If so consider adding this information as it is extremely relevant to this study as some of B. senegalensis could be misidentified due to the similarity of the species.

**Response 19** : We agree to the reviewer’s comment. We did not find any data focusing specifically on the distribution of these two species in Senegal. However, some publications predict their distribution according to environmental conditions (types of water bodies, rainfall).

This has been detailed in the introduction section - (Line 62/70).

I suggest you add some information about the MALDI-TOF MS as a research tool and its relevance to several species’ identification.

**Response 20** : We agree the reviewer's comment and more information on the relevance of using MALDI-TOF in the « Introduction » section – (Line 84/93).

Maybe you can add a sentence with the relevance of the present study at the end of the introduction.

**Response 21** : We agree with the reviewer's comment, we added a sentence to introduce the study in the introduction section - (Line 91).

Methods:

Since according to Hamlili FZ et. al, 2021 it is necessary a specific database depending on the storage condition and can impact the quality of the tool I suggest you indicate the storage condition performed in the present study. You can indicate that the methods were performed as Hamlili FZ et. al, 2021, but quickly describe it.

**Response 22** : We agree with this comment and have given more details on the method used with MALDI-TOF as described by Hamlili et al., 2021 (Line 128/155)

The same I can point to the other methods - genomic DNA extraction, nucleotide sequence-based analysis, Real-time-Polymerase Chain Reaction (RT-PCR), and PCR sequencing - readers need to know exactly the methods you performed (reproducible study).

**Response 23 :** We have corrected in the revised manuscript (Line 163/169 ; Line 171/178 ; Line 183/205)

Results:

What is the relevance of the MALDI-TOF MS technique if the identification differentiation between B. forskalii and B. senegalensis can be performed by molecular identification using the COI gene?

**Response 24** : We agree with the reviewer's comment, we addressed this issue in the "introduction" section of the study - (Line 79/91).

To improve the results view and comparison I think that characteristic MALDI-TOF MS spectra acquired from B. senegalensis feet should be added to the Figure 1 as a control.

**Response 25 :** Corrections have been made as recommended (Fig 3)

Can the infected snails be different from the non-infected under the MALDI-TOF MS analysis?

**Response 26** : We thank the reviewer for his question. This will be the subject of another paper, however, our preliminary work shows MALDI-TOF MS is capable of differentiating infected versus uninfected snails.

Reviewer #2 : Line 70 : « Desoxyribo » should be Deoxyribo

**Response 27 :** Corrections have been made as recommended - (Line 129)

Line 72 : « .. of Flight (MALDI-TOF MS) » : Consider « .. of Flight Mass Spectrometry (MALDI-TOF MS) »

**Response 28 :** Corrections have been made as recommended - (Line 130)

Line 31 : « Nucleotide sequence analysis identified 6 (11.0%), using COX1, and 3 (5.5%), using ITS2, S. hæmatobium, and 3 (5.5%) S. bovis » could be clarified to something to the effect of « Nucleotide sequence analysis identified 6 (11.0%) using COX1 and 3 (5.5%) using ITS2 for S. hæmatobium, and 3 (5.5%) using COX1 and 3 (5.5%) using ITS2 for S. bovis »

**Response 29 :** We agree with these comment and corrected the revised manuscript – (Line 29-31)

On line 32, the manuscript asserts that "This result is the first report of infection of B. forskalii by S. hæmatobium complex parasites." However, the study by Labbo and coworkers (reference 19) have previously reported B. forskalii with S. haematobium infection. The statement could be modified by appending "in Senegal" or otherwise rewritten to reflect that the current study, in contrast to the previous study, uses more precise methods to differentiate between B. forskalii and B. senegalensis in identifying B. forskalii infected with S. haematobium.

**Response 30 :** We agree with the reviewer’s remarks and we have clarified this sentence accordingly.

Reviewer #3: The paper is well written; no editorial suggestions.

--------------------

<b>Summary and General Comments</b></br></br>

Use this section to provide overall comments, discuss strengths/weaknesses of the study, novelty, significance, general execution and scholarship. You may also include additional comments for the author, including concerns about dual publication, research ethics, or publication ethics. If requesting major revision, please articulate the new experiments that are needed.

Reviewer #1: The present study is relevant since it is the first report of B. forskalii infected by S. hæmatobium complex parasites in Senegal. Although it improves the knowledge about host-parasite interactions it also highlights that more similar studies should be held in endemic sites to better understand the transmission of Schistosoma species. It is relevant that MALDI-TOF mass spectrometry analysis was applied again as a tool that allows the differential identification of B. forskalii and B. senegalensis snails since B. senegalensis is already one of the main intermediate hosts of S. hæmatobium.

Reviewer #2: -Summary of study

The manuscript describes a study of 55 B. forskalii snails with respect to S. haematobium and S. bovis infection in the delta region of the Senegal River in September 2020. The study addresses the challenge of differentiating B. forskalii and B. senegalensis by using MALDI-TOF MS and PCR sequencing (COX-Folmer primers) analyses and follows with a cercarial shedding test and PCR sequencing analyses for S. haematobium COX1 and ITS2, showing that B. forskalii of this region can carry S. haematobium, S. bovis, or possibly S. haematobium x S. bovis hybrid parasites.

-Strengths

The analysis methods (MALDI-TOF MS and PCR sequencing) increase the confidence that snail specimens represent B. forskalii, not B. senegalensis, and that these B. forskalii snails are carrying S. haematobium parasites.

-Weaknesses

Field sampling included a single region in Senegal during a limited temporal window in September 2020. Sampling of more regions over a larger temporal period would increase the confidence that B. forskalii, under a broader range of conditions and environments, can carry S. haematobium infection.

Reviewer #3: The authors' finding is important for the field of Schistosomiasis, particularly in endemic areas because they show definitively that Bu forskalli can host Sh. This finding will be of interest to parasitologists and malacologists; not only for the technology used to draw the study's conclusions, but also because of the implications for human infection. My "major revision" recommendation is mainly to address deficiencies in the Methods section, but does not necessarily require new experiments.
