# Supplementary material for: Identification of Bulinus forskalii as a potential intermediate host of Schistosoma hæmatobium in Senegal
Source: PLoS Negl Trop Dis. 2023 May 9;17(5):e0010584. doi: 10.1371/journal.pntd.0010584 (PMC10198482; doi:10.1371/journal.pntd.0010584)
Supplement: Attachment — Submitted filename: Reviewers Responses to questions.docx [file pntd.0010584.s005.docx]

Dear Editor,

Thank you for giving us the opportunity to submit an enhanced version of our manuscript. Please find below the list of our point to point responses to all reviewers’ comments, and a description of the changes we have made in the manuscript.

**Methods**-

Reviewer #1: Sentence lines 160-162 is duplicated on lines 163-166. (manuscript with track changes)

**Response 1** : Thanks for the remark, we have corrected this in the manuscript with track changes (Lines 163-168) and in the revised manuscript – (Lines 149-152)

Table 1 consider change it to Supplementary data.
**Response 2** : We agree with this comment and corrected the revised manuscript– (S1 Table)

Reviewer #2: (No Response)
Reviewer #4: The objetives are clearly articulated with a clear testable hypothesis stated
Sample size is somewhat small. This limits the statistical analysis.
Reviewer #5: 1- Line 100: Fig1 could not be downloaded in high resolution

**Response 3**: Thanks for the remark, we have corrected this in the manuscript – (Line 100)
2- Line 115: Please add the genomic coordinates of the area in which the snails were sampled

**Response 4**: We agree with the reviewer's comment, we have thus accordingly added the geographical coordinates in the revised manuscript - (Lines 116-118).

3- Line 119: Please make it clear which morphological features of the *Bulinus forskalii* snail species were analyzed

**Response 5**: We agree with the reviewer's comment, we have added these elements in the revised manuscript - (Line 113-115 ; 120-122).
4- Line 145: sentence is duplicated, needs correction

**Response 6**: Thanks for the remark, we have corrected this in the manuscript – (Line 149-152)
5- Table 1 needs correction: some terms are truncated (Ethanol absolu) or incorrect (Coordonates), etc

**Response 7**: We have corrected this in the revised manuscript – (S1 Table)
6- Line 180 : please specify what has been used as negative and positive controls

**Response 8**: Thank you for your suggestion, we have corrected this in the manuscript – (Line 183-184)

--------------------
**Results**

Reviewer #1: Line 264 change paratised to parasitized. (manuscript with track changes)

**Response 9**: Thanks for the remark, we have corrected this in the manuscript with track changes (Line 265) and in the revised manuscript (Line 240)
Why on table 3 do you only have 10 specimens identified on the table ? On lines 259 - 260 you indicate that 13 were positives through shedding and 17 through RT-PCR.

**Response 10**: We agree with the reviewer's comment.

Of the 55 *B. forskalii* specimens, 13 (23.6%) or 17 (30.9%) were infected with *Schistosoma* parasites, using the cercarial shedding test or the Dra1-RT PCR, respectively. We performed both tests on the same samples (n=55), first the cercarial excretion which proved to be less sensitive here, because cercariae are excreted only at their maturity, whereas Dra1-PCR is able to detect the parasite before, i.e. at the lower stages of its development (sporocysts or redia). These distinct condition explains our apparently discrepant results: 13 (23.6%) for cercariae emission and 17 (30.9%) for Dra1-PCR. Among the 17 specimens positive to *S*. *haematobium* complex specific RT-PCR-Dra1, only the 10 could be identified by sequencing, the others had no sequences or sequences of poor quality and were removed from our analysis.

Reviewer #2: (No Response)
Reviewer #4: (No Response)
Reviewer #5: The quality of the figures should be improved.

**Response 11**: We agree with the reviewer's comment, corrections have been made as recommended.

Reviewer #1: Line 283 change paratised to parasitized. (manuscript with track changes)

**Response 12**: We agree with this comment and corrected in the manuscript with track changes – (Line 283) and in the revised manuscript – (Line 251).

Reviewer #2: (No Response)

Reviewer #4: (No Response)

Reviewer #5: Line 250: Has the authors tried/identified sporocystes in the collected *Bulinus forskalii* snails?

**Response 13:** We thank the reviewer for this question. We did not try to identify sporocysts on *B. forskalii,* which requires crushing the snail specimen and doing the microscopical analyses between slide and coverslip. This could be interesting to support the cercarial emission test, but identification of the sporocysts requires a great expertise. However, we have identified schistosome cercariae based on their morphological appearance.

--------------------

Reviewer #1: (No Response)

Reviewer #2: (No Response)

Reviewer #4: (No Response)

Reviewer #5: (No Response)

--------------------
**Summary and General Comments**

Reviewer #1: I suggested minor revision just to adjust some details and regarding the Table 3 that I still didn’t understand why only consider 10 specimens to add on the table 3.

(Please refer to our response N°10)

Reviewer #2: The authors have adequately addressed the reviewers' comments and have improved the quality of the manuscript in this revision. The manuscript is suitable for publication.

Reviewer #4: The paper presented by Mouhamadou et al, entitled Identification of Bulinus forskalii as a potential intermediate host of Schistosoma haematobium in Senegal, describes a field study for the collection and identification of *B. foskalii* snails for infection with *S. haematobium* and *S. bovis* in an area of the Senegal River during the month of September 2020. The finding that *B. forskalli* snails may be intermediate hosts of *S. haematobiun* is of great interest for the control of schistosomiasis in Sengegal.
The manuscript is well written and structured and allows a good understanding.
Methodologically it is correct, but I would like the authors to discuss in more depth the advantages of the MADI-TOF MS technique for the identification of snails, taking into account that they have previously been identified without problems by morphological characters. They comment in the introduction that morphological identification requires a great deal of expertise, that DNA identification is time-consuming and that there is not much information in databases. In schistosomiasis endemic areas, is it feasible to make a MALDITOF ? It also requires knowledge and experience to do it, as well as the databases to make a good comparison of identifications. Please try to explain this in more detail. Maybe there are not so many advantages compared to an identification of snails by morphology.

**Response 14**:	We agree with the reviewer's comment and we have further discussed the benefits of using MALDI-TOF MS in the discussion section of the manuscript – (Line 271 - 281)

The use of MALDI-TOF MS is not yet recognized in endemic areas; but it may be routinely used in the near future. This tool is already available in Dakar, Senegal, although schistosomiasis is present in all regions of the country except in the Dakar area (Senghor et al., 2015). Its use does not require much skills and especially only limited malacological expertise for the identification of snails in the context of schistosomiasis. Databases are increasingly available online and setting up an internationally accessible MALDI-TOF MS spectra database may become feasible in the near future.
Typo in Line 188; *S. bovis* should be written in italics.
**Response 15**:	Thanks for the remark, corrections have been made as recommended - (Line 192)
Reviewer #5: The authors provide a report that is centered around the identification/description of *Bulinus forskalii* snails that can host *S. hæmatobium* in a limited region of the Senegal River. The manuscript has been greatly improved after the first revision: the descriptions are clearer, the results better presented now, and additional discussions improved the interpretations. I have two main concerns	:

1- the fact that a single, limited region in Senegal was sampled in a short temporal window (September 2020). Additional sampling in other regions over a larger period could strengthen the authors’ claim.

**Response 16**: We agree with the reviewer's comment, this will be developed in further publication that will also detail the contribution of *B. forskalii* as an intermediate host of *S. haematobium* as part of a larger sampling with other *Bulinus* species.
2- a demonstration that *Bulinus forskalii* can be infected by *S. hæmatobium* outside the field, in the laboratory could strengthen the manuscript. Has this been already performed, or is it possible ?

**Response 17**: We did not have the possibility to perform an experimental infection on *B. forskalii*. However, in our bibliographic research, Labbo *et al.* 2007 in their work succeeded in an experimental infection of *B. forskalii* from Niger with *S. haematobium* originating from the same region.

Other points that should be considered are below:
Introduction
1- Line 77: Replace “exclude” by “excluded”

**Response 18**: Thanks for the remark, corrections have been made as recommended - (Line 77).
--------------------

Reviewer #1: No
Reviewer #2: Yes: Kenji Ishida
Reviewer #4: No
Reviewer #5: No
